# Taylor–Couette and related flows on the centennial of Taylor’s seminal *Philosophical Transactions* paper: part 1

**DOI:** 10.1098/rsta.2022.0140

**Published:** 2023-03-20

**Authors:** Richard M. Lueptow, Rainer Hollerbach, Eric Serre

**Affiliations:** ^1^ Department of Mechanical Engineering, Northwestern University, Evanston, IL 60201, USA; ^2^ School of Mathematics, University of Leeds, Leeds LS2 9JT, UK; ^3^ M2P2, Aix Marseille University, Cent. Marseille, CNRS, Marseille, France

**Keywords:** Taylor–Couette flow, stability analysis

## Abstract

In 1923, the *Philosophical Transactions* published G. I. Taylor’s seminal paper on the stability of what we now call Taylor–Couette flow. In the century since the paper was published, Taylor’s ground-breaking linear stability analysis of fluid flow between two rotating cylinders has had an enormous impact on the field of fluid mechanics. The paper’s influence has extended to general rotating flows, geophysical flows and astrophysical flows, not to mention its significance in firmly establishing several foundational concepts in fluid mechanics that are now broadly accepted. This two-part issue includes review articles and research articles spanning a broad range of contemporary research areas, all rooted in Taylor’s landmark paper.

This article is part of the theme issue ‘Taylor–Couette and related flows on the centennial of Taylor’s seminal *Philosophical Transactions* paper (part 1)’.

## 1. Taylor’s seminal 1923 paper

The geometric simplicity of fluid flow betweendifferentially rotating concentric cylinders has attracted the interest of scientists for centuries, including Isaac Newton and George Stokes [1]. However, it was G. I. Taylor who first connected theory and experiment in his seminal 1923 paper in the *Philosophical Transactions A* [2]. His ground-breaking investigation is considered by many as convincing proof of not only the applicability of mathematical approaches to predict stability but also the fundamental correctness of the Navier–Stokes equations and the no-slip boundary condition, thereby firmly establishing the foundation for these fundamental concepts that are crucial to modern fluid mechanics.

Over the past century, this single paper has inspired a broad range of research topics, so much so that Taylor–Couette flow has become an enduring metaphor for fundamental studies in nonlinear dynamics, stability analysis, pattern formation, centrifugal flows, transitional flows, Görtler/Dean vortices and general vortical flows. The attraction of Taylor–Couette flows is its beautiful vortical patterns that are easily generated and visualized. Equally important is the broad range of disciplines connected to one another via Taylor–Couette flow including physics, mathematics and engineering. As a result, Taylor–Couette flow has become a test bed for hypotheses related to stability, drag reduction, astrophysical and geophysical flows and fundamental aspects of turbulence. Not only that, the flow has been used in practical engineering problems such as mixing and filtration, among other applications. Add to this, the richness of the topic, which connects it to an expansive range of canonical flow geometries, questions of stability, physics of vortical flows and other key aspects of fluid dynamics.

Taylor’s original paper is remarkable in many ways, and its importance in the field of fluid mechanics becomes evident when reading the first few paragraphs of the paper. Taylor begins by noting that in experiments of the flow of fluids past solid boundaries, ‘steady motion breaks down and eddying flow sets in’ [2, p. 289], and that ‘A great many attempts have been made to discover some mathematical representation of fluid instability, but so far they have been unsuccessful in every case’ [2, p. 289]. Taylor goes on to enumerate attempts by giants in the field of fluid mechanics including Kelvin, Rayleigh, Sommerfeld, Orr, Mises and Hopf to examine flow between two infinite parallel plates, but who all concluded that ‘the fundamental small disturbances of this system are stable’ [2, p. 289]. Taylor also discusses [2, p. 290] Osborne Reynolds’ (1895) [3] and William Orr’s (1907) [4] ‘promising’ but unsuccessful attempts to consider the stability related to Reynolds’ classic 1883 experimental results [5] for the transition to turbulent flow in a pipe.

Clearly, Taylor saw this as a challenge. In fact, he quotes Orr’s remarks that ‘It would seem improbable that any sharp criterion for stability of fluid motion will ever be arrived at mathematically’ [4, p. 75], [2, p. 290]. Taylor proves just the opposite by intentionally considering flow between concentric rotating cylinders rather than between two parallel planes. Taylor made clear that he was searching for an example where a mathematical representation could be found to match observable results, and he ended up focusing on Rayleigh’s instability criterion [6, p. 210] ‘so that a detailed comparison can be made between the results of analysis and those of experiment’ [2, p. 290]. The motivation was simply that ‘It is very much easier to design apparatus for studying the flow of fluid under pressure through a tube, or the flow between two concentric rotating cylinders’ [2, p. 291]. Taylor then opts for studying the concentric cylinder case rather than pipe flow because he notes that in pipe flow small disturbances are stable while large disturbances, which are much more difficult to study, are unstable [2, p. 291].

A remarkable aspect of Taylor’s paper is the quality and thoroughness of the meticulous experiments that he performed. In their treatise on hydrodynamic stability, P. G. Drazin and W. H. Reid point out that the work ‘was remarkably complete experimentally as well as theoretically’ [7, p. 104]. Taylor notes that Maurice Couette had considered a rotating outer cylinder in his attempts to measure fluid viscosity [8] and that the sharp change in drag above a certain rotational velocity of the outer cylinder could be ‘attributed to a change from steady [laminar] to turbulent motion’ [2, p. 292]. However, it was Arnulph Mallock who in another *Philosophical Transactions* paper in 1896 [9] found the centrifugal instability that occurs when the inner cylinder rotates with the outer cylinder at rest, which Taylor notes [2, p. 292] ‘is in accordance with Lord Rayleigh’s theoretical prediction for the case of an inviscid fluid’ [10]. Apparently, though, Mallock did not consider low enough rotational speeds to observe stable flow with the inner cylinder rotating [1], which Taylor points out [2, p. 292] Rayleigh had proven must occur for a viscous fluid [11].

Taylor’s experimental apparatus appears to have been quite elegant, with Taylor having diligently corrected several of the weaknesses that plagued Mallock’s experiments. Taylor designed an apparatus in which either cylinder could be rotated with a length-to-gap aspect ratio as high as nearly 400 to minimize endwall effects. Ball bearings and heavy iron supports were used to assure the integrity of the apparatus. The outer cylinder was precision bored, ground and polished glass so as to use ‘the method employed by Osborne Reynolds … to inject a thin continuous stream of colouring matter,’ [2, p. 330] a neutrally buoyant fluorescene dye. Using this apparatus, Taylor could directly compare the measured rotational speed for transition to unstable flow and the subsequent vortex spacing to his theoretical stability prediction as well as photograph the vortex structure.

Another remarkable aspect of the paper is the degree to which the experiments connected with the mathematical results for linear stability. In fact, Drazin and Reid comment that ‘the closeness of the agreement between his theoretical and experimental results was without precedent in the history of fluid mechanics’ [7, p. 105]. In fact, Taylor’s stability analysis and experiments get to the root of the equations of motion for a viscous fluid that were formulated by Claude-Louis Navier [12] and George Gabriel Stokes [13], which we now know as the Navier–Stokes equations. A crucial issue that arose in the late 1800s was the measurement of the fluid viscosity, which is precisely the problem that Couette and Mallock were considering experimentally in their carefully designed concentric, differentially rotating cylinder apparatuses [8,9]. Of course, it was the Taylor instability that made the results for these measurements confusing. When the inner cylinder rotates with the outer cylinder stationary the centrifugal instability appears only above a certain rotational speed, while for the outer cylinder rotating it is the viscous instability leading to turbulence that appears. Another question at the time was the appropriate boundary conditions to use in the mathematical analysis of fluid flow. Physicist Russ Donnelly noted ‘that Stokes is concerned that the boundary conditions at the solid surfaces are unknown’ and that ‘nearly a century would elapse [from Stokes' 1845 work] before the no-slip condition for a fluid at a solid wall was universally accepted.’ Donnelly goes on to say that ‘Indeed, it was Taylor’s analysis of rotating cylinder flow that settled the matter’ [1, p. 34]. The success of Taylor’s analysis in matching his meticulous experiments ‘was taken by many as perhaps the most convincing proof of the correctness of the Navier–Stokes equations and of the no-slip boundary condition’ [1, p. 37].

G.I. Taylor’s influence on the study of fluid dynamics was immense. George Batchelor, a brilliant fluid mechanician in his own right, called G.I. Taylor ‘one of the greatest of the ‘masters’ in fluid mechanics’ [14, p. 1]. He noted three principal features of Taylor’s contributions: ‘First, they show profound insight and ability to see how things work physically; secondly, they have the elegance and beauty that is conferred by functional simplicity, simplicity of experimental design and simplicity of mathematical argument, both being sufficient, and no more than sufficient, for the purpose in hand; and thirdly, and most important, they exhibit that uncanny knack common to the greatest scientists of recognizing the essential aspects of a phenomenon or a problem that everyone will see later to be significant and of wide applicability’ [14, pp. 1, 3]. Indeed, this is the case for Taylor’s 1923 paper on the stability of what we now call Taylor–Couette flow, honouring both Taylor, who solved the problem, and Couette, whose original experiments inspired Taylor to focus on the flow in the annulus between differentially rotating cylinders. Taylor’s choice of the problem and the stability analysis approach demonstrate extraordinary insight; his analysis and experiment are elegant because of their simplicity and execution; and his analysis is based on the minimal essential aspects of the problem, which have turned out to be significant because they can be so widely applied.

Taylor’s contributions are so broad and influential that an entire graduate course in modern classical physics was developed based solely on Taylor’s contributions [15]. In the course, the first topic that Michael Brenner and Howard Stone consider in detail is Taylor’s 1923 paper. They point out that the concept of stability had been previously formulated, but no calculation agreed with experiment. The focus in the course is not only on the linear stability analysis itself but also that it ‘demonstrated unambiguously that both the approach used in the stability calculation, and its underlying assumptions (the boundary conditions), were correct’ [15, p. 32]. Equally important in this course was Taylor’s motivation to find a problem in which the mathematical analysis agreed with experiments, again pointing to the influence of this single paper in modern classical physics.

Even Taylor’s closing remarks in his 1923 paper display remarkable insight into the future of research for this now canonical flow system. For example, he notes the variety of nonlinear states that can appear in a Taylor–Couette cell including spiral vortices, wavy vortices and turbulence, all of which have proven to be important research topics over the past century. Perhaps physicist Richard Feynman sums it up best in his famous lectures, this one on ‘The Flow of Wet Water,’ where in the context of Taylor’s 1923 paper he says: ‘the main lesson to be learned from [Taylor’s analysis] is that a tremendous variety of behaviour is hidden in the [Navier–Stokes equations]. All the solutions are for the same equations, only with different values of [the Reynolds or, equivalently, Taylor number]. We have no reason to think that there are any terms missing from these equations…. That we have written an equation does not remove from the flow of fluids its charm or mystery or its surprise.’ [16] (as noted in [15, p. 33]). Indeed, charm, mystery, and, often, surprise permeate the study of Taylor–Couette flow over the century since Taylor’s seminal paper.

## 2. Topics in this theme issue

Part 1 of this theme issue is a combination of review articles and research articles having a root in Taylor’s 1923 paper and often connecting directly to Taylor’s vision of future work on the problem. The authors who have contributed to this theme issue are leading researchers in the field of Taylor–Couette and related flows. They represent an international community of scientists and engineers with research interests that span a broad range of flow physics and applications, all of which can trace their heritage back to Taylor’s 1923 paper in the *Philosophical Transactions*.

For the classical Taylor–Couette problem with a Newtonian fluid, the focus of modern research has largely moved beyond the linear onset of instability studied by Taylor to consider instead the highly supercritical, turbulent regime. Crowley *et al.* investigate ‘a dynamical skeleton of turbulence’ experimentally and numerically. Wiswell *et al.* conduct experiments on end-effects in low aspect ratio Taylor–Couette flow. Jeganathan *et al.* present numerical results on the origin of turbulent Taylor rolls. Oishi & Baxter use a generalized quasi-linear approximation to study non-normality in spiral turbulence.

Fundamentally new areas of research are opened up by combining Taylor–Couette flows with convection. Kang *et al.* and Meyer *et al.* each present numerical results obtained by imposing radial temperature gradients in the underlying cylindrical geometry. Further extensions of Taylor–Couette flow include multi-phase flows, where Baroudi *et al.* review Taylor–Couette flow of suspensions, followed by experimental studies by Alam & Ghosh, Yi *et al.* on emulsions, and Blaauw *et al.* on bubbly drag reduction by switching from fresh to salt water.

Three further extensions of the classical Taylor–Couette problem are magnetohydrodynamic, ferrofluidic and viscoelastic flows. At first glance, these might seem very different, but they modify the original problem in somewhat similar ways, by providing new ways of coupling fluid parcels via magnetic tension in magnetohydrodynamics, magnetic forces in ferrofluids, or polymeric elasticity in viscoelastic flows. Guseva & Tobias present numerical studies and theoretical analysis of transition to chaos and modal structure in magnetohydrodynamic flows. Altmeyer numerically explores ferrofluidic wavy Taylor vortices under alternating magnetic fields. For the viscoelastic problem, Boulafentis *et al.* review experiments involving elasto-inertial transitions, and Song *et al.* review turbulent flows of dilute polymeric solutions.

Finally, there are a variety of systems and geometries that are not strictly Taylor–Couette flows as such, but are nevertheless closely related. Martinand *et al.* review routes to turbulence in rotating disc boundary layers and cavities, demonstrating that ideas and methods similar to those used in the Taylor–Couette geometry can often be applied in other flow systems as well.

## 3. Conclusion

Although Taylor–Couette flow has been studied for a century (as well as long before Taylor put its fundamental essence on a solid foundation), it continues to provide a basis for a broad range of research. This two-part issue of the *Philosophical Transactions* builds on the ongoing interest in Taylor–Couette flow and its many important derivatives in terms of current research, perspectives on the influence of Taylor’s seminal paper, and its future impact on many related fields.

## Data Availability

This article has no additional data.
